# Terrestrial Hot Spring Systems: Introduction

**DOI:** 10.1089/ast.2018.1976

**Published:** 2019-12-04

**Authors:** David J. Des Marais, Malcolm R. Walter

**Affiliations:** ^1^ Exobiology Branch, NASA Ames Research Center, Moffett Field, California, USA.; ^2^ Australian Centre for Astrobiology, School of Biological, Earth and Environmental Sciences, University of New South Wales, Sydney, Australia.

**Keywords:** Sinter, Yellowstone, Mars, Taphonomy, Biosignatures

## Abstract

This report reviews how terrestrial hot spring systems can sustain diverse and abundant microbial communities and preserve their fossil records. Hot springs are dependable water sources, even in arid environments. They deliver reduced chemical species and other solutes to more oxidized surface environments, thereby providing redox energy and nutrients. Spring waters have diverse chemical compositions, and their outflows create thermal gradients and chemical precipitates that sustain diverse microbial communities and entomb their remnants. These environments probably were important habitats for ancient benthic microbial ecosystems, and it has even been postulated that life arose in hydrothermal systems. Thermal spring communities are fossilized in deposits of travertine, siliceous sinter, and iron minerals (among others) that are found throughout the geological record back to the oldest known well-preserved rocks at 3.48 Ga. Very few are known before the Cenozoic, but it is likely that there are many more to be found. They preserve fossils ranging from microbes to trees and macroscopic animals. Features on Mars whose morphological and spectroscopic attributes resemble spring deposits on Earth have been detected in regions where geologic context is consistent with the presence of thermal springs. Such features represent targets in the search for evidence of past life on that planet.

## 1. Introduction

**FIG. 1.**
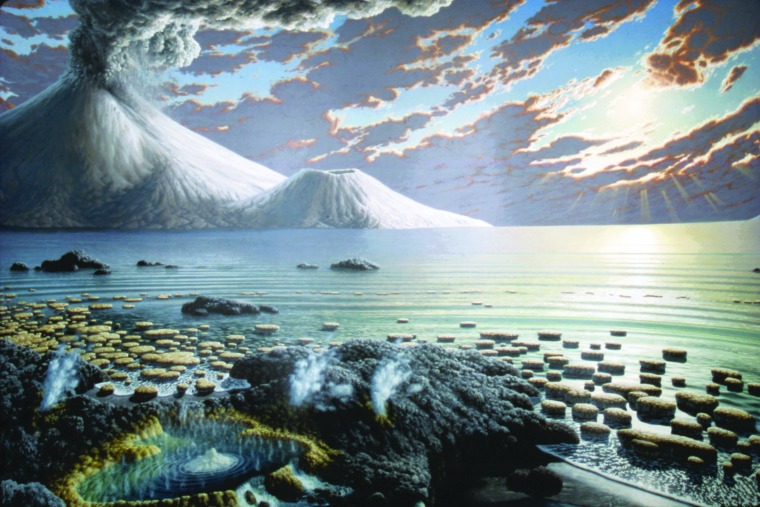
Artist's concept of Earth 3.5 billion years ago. Reproduced with permission of Smithsonian Institution.

Terrestrial hot springs harbor diverse and abundant microbial communities. They are the “tropical rain forests” of the microbial world. They are also potent chemical reactors and the sites of profuse mineral deposition that preserves those communities into the geological record. Many springs are the surface manifestations of subterranean “epithermal” mineral deposits and as such are exploration targets for gold in particular. Consequently, they are of interest to both biologists and geologists, and their history has been traced back to the oldest well-preserved rocks on Earth (Fig. 1). For all these reasons, they are also a prime target in the search for both extant and ancient life elsewhere in the Solar System, particularly on Mars. They are central to many exploration models.

This introductory review summarizes the attributes that enable terrestrial hot spring systems to sustain microbial communities and create a fossil record. These systems can be dependable water sources, even in arid environments. They deliver reduced chemical species and other solutes to more oxidized surface environments, thereby providing redox energy and nutrients. Spring waters have diverse chemical compositions, and their outflows create thermal gradients and chemical precipitates that sustain diverse microbial communities and entomb their remnants. Accordingly, hot spring systems preserve fossils ranging from microbes to trees and macroscopic animals.

Other papers in this special collection provide much more detail, including reviews of diverse hot spring systems outside of North America.

Where heat and water meet, there are likely to be hot springs. Most often this is in volcanic regions, for example, the Yellowstone caldera in the United States, the Taupo Volcanic Zone in New Zealand, Iceland, and the Kamchatka Peninsula in Russia. Such springs also occur where there are deeply circulating artesian waters in sedimentary basins such as the Great Artesian Basin in Australia (Habermehl, 1982). Ephemeral springs also occurred at the sites of impact of large meteorites such as at Haughton Crater in Canada (Osinski *et al.,* 2001). Springs often occur in and around lakes in all of these environments. Here we confine our discussion to springs in volcanic environments. In order to introduce extant examples, we have concentrated on springs in Yellowstone National Park (YNP) because they are chemically diverse and particularly well known. Other papers in this special collection address additional hot spring systems.

Baross *et al.* (National Research Council, 2007) have identified the following potentially universal attributes of life: (1) Life exploits thermodynamic disequilibrium in the environment in order to perpetuate its own disequilibrium state. (2) Life as we know it consists of interacting sets of covalently bonded molecules that include a diversity of heteroatoms (*e.g.,* N, O, P, S, etc. in Earth-based life) that promote chemical reactivity. (3) Life requires a liquid solvent that supports these molecular interactions. (4) Life employs a molecular system capable of Darwinian evolution. Hot spring environments support each of these attributes in the following ways (Walter and Des Marais, 1993): (1) Hot spring waters typically deliver reduced chemical species to more oxidized surface environments, thus providing biochemically useful sources of redox energy (Shock *et al.,* 2005). (2) Thermal spring environments provide all the essential chemical building blocks of life. (3) Thermal springs can be dependable water sources, even in arid environments. (4) Hydrothermal systems can sustain environmental conditions favoring the persistence of the molecular mechanisms that enable self-replication and evolution (Des Marais, 2013).

Hot springs have long been considered as potential cradles for the origins of life, but until recently the focus has been on the very high temperature submarine “black smokers.” Recently, however, attention has shifted to terrestrial settings for a number of reasons but particularly because the lower temperatures are less destructive of organic molecules and the characteristic wetting and drying cycles are conducive to the polymerization of large molecules (Damer and Deamer, 2015; Deamer and Georgiou, 2015; Van Kranendonk *et al.,* 2017; Westall *et al.,* 2018).

With regard to the search for life elsewhere, rational exploration requires a guiding hypothesis, an “exploration model.” Geologists are particularly familiar with this approach in the search for precious metals and petroleum. In the search for life beyond Earth, we have only one example to work from—life here. The focus on terrestrial hot springs results from the logic expressed above. The presence of volcanoes and water ice on Mars has been known since the 1960s, with images from the Mariner missions of NASA. It follows from these and from later observations that there must have been hot springs at least early in the history of Mars, when the volcanoes were active. Currently active springs were long ago proposed as targets in the search for extant life on Mars (Lederberg and Sagan, 1962), but in all subsequent exploration, no active springs have been detected (with the possible exception of some “slope lineae”). A focus on ancient springs was proposed by Walter (1988), Walter and Des Marais (1993), Walter (1999), and Allen and Oehler (2008), and candidate sites are now recognized and are discussed below.

In the sections below, we expand on some of these observations. Many are considered in much more detail in the papers that follow in this collection.

## 2. Basic Geochemistry of Hot Spring Deposits

The compositions and geometries of hot spring deposits are influenced by the compositions of ascending hydrothermal fluids, the spring environments, and the processes associated with sinter deposition.

Hydrothermal fluids and their deposits can be grouped into end-member categories (Drake *et al.,* 2014). *Alkaline chloride fluids* form when groundwater and volcanic gases (*e.g.,* CO_2_, H_2_S) interact with silicate rocks. Upon cooling, these fluids typically form siliceous sinter spring deposits (opal-A: SiO_2_·nH_2_O) (White *et al.,* 1964). *Acid-sulfate fluids* form when ascending H_2_S is oxidized to form H_2_SO_4_ which then reacts with surrounding rocks, creating alteration minerals such as clays, oxides, and silica residues (*e.g.,* Schinteie *et al.,* 2007). Acid sulfate fluids also can mix with alkali chloride fluids and form acid sinters, but these deposits are generally less common and thinner than deposits derived principally from alkaline chloride fluids (Drake *et al.,* 2014). *Bicarbonate-rich fluids* arise when volcanic gases and groundwater interact with carbonate rocks, and they can create travertine (CaCO_3_) deposits (Fouke *et al.,* 2000). *Iron-rich springs* harbor microbial communities (Parenteau and Cady, 2010) and create abundant flocculant iron-bearing deposits.

The processes associated with the precipitation of siliceous versus carbonate sinters are quite different and thus create distinctively different deposits. Emergent alkali chloride waters cool and become supersaturated with respect to silica because its solubility is strongly temperature dependent (White *et al.,* 1964). Silica proceeds through a multistep sequence to form dimers, polymers, and colloids (Chan, 1989) that ultimately accrete on surfaces to build sinter deposits. Accordingly, siliceous sinters form low broad spring mounds that can extend tens of meters or more from their springs. In contrast, emerging bicarbonate-rich fluids degas CO_2_ at or near the spring and immediately become highly supersaturated with respect to calcium carbonate. Travertine deposition is fastest near the springs and typically creates high-relief structures (*e.g.,* Fouke *et al.,* 2000) that generally do not extend as far from the springs as siliceous sinters.

## 3. Biogeochemistry of Modern Hot Spring Systems

Modern thermal spring systems offer multiple insights about the microbial ecosystems they sustain. They reveal thermal tolerances of diverse groups of organisms and how microbial community structures vary along thermal gradients. These systems provide examples of microbe-mineral interactions and the associated effects on microbial taphonomy. Modern systems present an array of microbial biosignatures whose remnants we should seek to discover and interpret in ancient thermal spring deposits. Yellowstone National Park (YNP) provides excellent examples of a broad diversity of hot spring systems, microbial communities, and an abundant literature of research on their attributes. The following few examples of this work highlight some particularly important insights gained from hot spring ecosystems.

### 3.1. Energy sources

Hot springs provide abundant and diverse sources of energy for microbial ecosystems. As noted above, a potentially universal attribute of life is that it “…exploits thermodynamic disequilibrium in the environment in order to perpetuate its own disequilibrium state” (National Research Council, 2007). Terrestrial hot springs create nutrient-rich oases that sustain photosynthetic communities, even when their surrounding environments are quite extreme (*e.g.,* Fernandez-Turiel *et al.,* 2005). Life also thrives in hot springs even where darkness prevails or where elevated temperatures (>74°C, Castenholz, 1969) preclude photosynthesis. The biological mediation of reactions between hydrothermal fluids and geological materials provides a variety of chemical energy sources for microbial communities. Shock *et al.* (2005) calculated the energy yields for an extensive array of redox reactions involving reduced hydrothermal fluids under the conditions in Obsidian Pool, YNP. The yields depend strongly upon particular electron acceptors in the following list from the highest to lowest energy yields: O_2_, nitrate, nitrite, elemental S, magnetite, hematite, goethite, sulfate, CO, and bicarbonate/CO_2_. A key perspective from this study is that many hot spring environments can receive light and provide diverse sources of chemical energy that, in turn, can sustain diverse microbial communities under remarkably favorable conditions.

### 3.2. Alkaline chloride springs

**FIG. 2.**
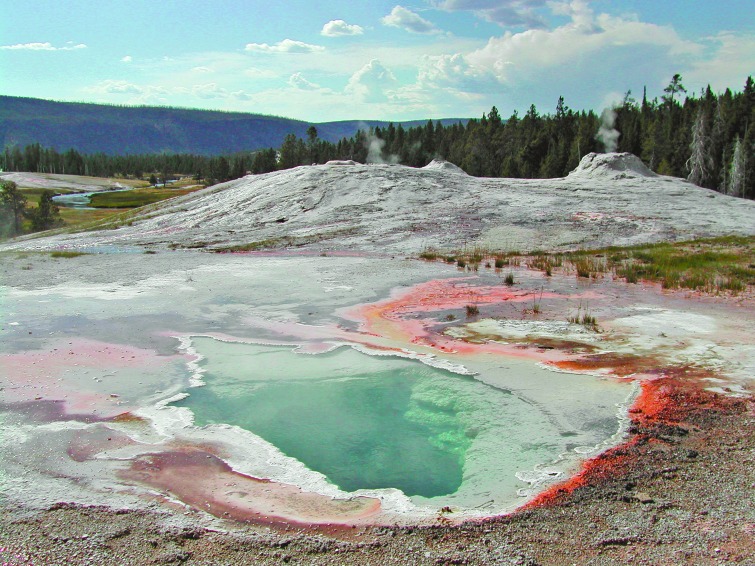
Siliceous sinter deposits surrounding Lion Group geysers and Doublet Pool, Upper Geyser Basin, Yellowstone National Park, Wyoming, USA.

**FIG. 3.**
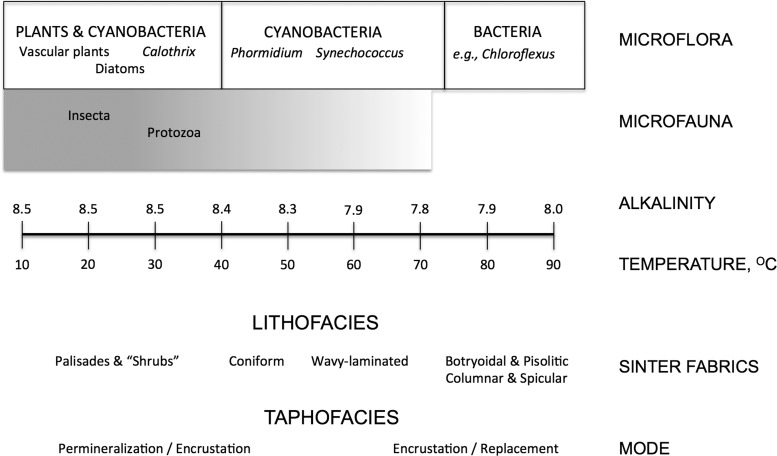
Environments and facies of siliceous alkaline springs based on observations in Yellowstone National Park, USA. Modified after Cady and Farmer (1996).

Benthic microbial communities thrive in the silica-depositing alkaline chloride hot springs in YNP (*e.g.,* Fig. 2). Cyanobacteria dominate the communities at temperatures up to 73°C but are not found at higher temperatures (Walter, 1976b; Walter *et al.,* 1976; Cady and Farmer, 1996). Morphologically distinct fabrics occur as stromatolites at the lower temperatures, but above 73°C the sinter was first interpreted as abiotic (Walter, 1976a), despite the presence (Brock, 1978) of high-temperature bacteria and archaea. Later work (Cady and Farmer, 1996; Fig. 3) using electron microscopy identified microbial biofilms in the high-temperature columnar and spicular geyserite that forms in the splash zones around hot pools and geysers. The biofilms appear to contribute to the morphology of the geyserite. For example, biofilms are thicker on the tips of spicules. At all temperatures silica precipitates on the individual microbes forming molds of the filaments and unicells. Open spaces and pores are later infilled by silica during early diagenesis.

Alkaline chloride springs have received the most attention regarding their microbial ecology and taphonomy. These springs were the focus of pioneering research on thermophilic extremophiles (*e.g.,* Brock and Freeze, 1969; Brock, 1978; Castenholz, 1969). Subsequent analyses of 16S rRNA revealed that these communities are remarkably diverse (*e.g.,* Ward *et al.,* 1990, 1998), despite the seemingly harsh conditions.

Patterns of diversity within hot spring microbial mats have been related to the ecology of these communities and how they have adapted to environmental gradients in hot spring systems (*e.g.,* Ward and Cohan, 2005). For example, populations of *Synechococcus* cyanobacteria exhibit closely related rRNA sequence variants whose differences are discretely distributed along transects that run parallel to the flow of the spring channel. This relationship is consistent with the interpretation that these distinct populations represent adaptations to different temperatures. Smaller yet discernible genetic differences among distinctly pigmented *Synechococcus* cyanobacteria with depth in the mats potentially indicate acclimation to different microenvironmental conditions. These and additional observations hint that genetic variations arose through adaptive radiation in these communities (Ward and Cohan, 2005). Hot spring mats also provide opportunities to understand the nature and extent of horizontal gene transfers within microbial communities.

Thiel *et al.* (2017) characterized the diversity and metabolic functions of microbial populations situated at various depths within mats at Octopus and Mushroom Springs. Populations of phototrophs and chemotrophs in the under-mat region utilized inorganic carbon via all four bacterial carbon fixation pathways as well as organic compounds derived from various cellular components and fermentation products of the community. Other genes were identified indicating nitrogen fixation, sulfate reduction and sulfide oxidation, as well as hydrogen production and consumption within the mats.

These and a host of other research findings indicate that hot spring microbial mats are diverse and complex and that conceivably they created a rich fossil record that has been preserved within ancient hot spring sinters that await our discovery.

### 3.3. Acid sulfate springs

Acid sulfate and acid sulfate chloride hot springs exhibit pH values typically <4, causing their microbial communities to differ substantially from communities in more alkaline spring waters. For example, cyanobacteria are key primary producers in more alkaline springs but are essentially absent in acid sulfate springs. The predominant dissolved species in Norris Geyser Basin acid sulfate chloride spring waters include Na^+^, Cl^-^, SO_4_^2-^, H^+^, Fe^2+^, As^3+^, H_2_S, CO_2_, CH_4_, and H_2_ (Inskeep and McDermott, 2005). These investigators documented distinct trends in geochemical zonation and microbial communities arrayed along the outflow channels. Proximal to the springs, sulfide oxidation created elemental sulfur deposits that could serve as electron acceptors in redox reactions involving H_2_ or organic carbon. Analyses of 16S rDNA sequences of mats in this zone indicated that the archaea *Stygiolobus* sp., *Thermocladium* sp., *Caldococcus* sp., *Caldisphaera* sp., *Thermocaldium* sp., and the bacterium *Desulfurella* sp. were the closest cultivated relatives of microorganisms in these springs that might mediate these reactions (Inskeep and McDermott, 2005). The bacterium *Hydrogenobaculum* sp. was the closest relative of microorganisms that could mediate redox reactions involving H_2_, H_2_S, S^o^, As^3+^, and O_2_. Farther downstream, elemental sulfur deposits waned, and hydrous iron oxides with associated As^5+^ became prominent due in part to increased dissolved O_2_. Possible redox reactions include O_2_ coupled with organic carbon and reduced As and Fe species, and Fe^3+^ coupled with organic carbon. Analyses of 16S rDNA sequences of mats in this zone indicated that the bacteria *Hydrogenobaculum* sp., *Desulfurella* sp., *Acidimicrobium* sp., *Thiomonas, Meiothermus* sp., and *Marinithermus* sp. and the archaea *Metallosphaera* were the closest cultivated relatives of microorganisms in these springs that might mediate these reactions. Also, multiple clones were identified whose closest relatives were uncultured archaeal and bacterial sequences (Inskeep and McDermott, 2005).

Cyanidiales is an order of unicellular red algae that is a dominant component of microbial communities inhabiting acidic hot springs, soils, and endolithic habitats (Brock, 1978). Accordingly, Cyanidiales holds the potential for preservation in siliceous sinters in these springs. Skorupa *et al.* (2013) documented a direct relationship between moisture and the habitat preference and viability of Cyanidiales populations in YNP. For example, they observed both the *Galdieria* and *Cyanidioschyzon* lineages of Cyanidiales in soil and endolithic habitats, whereas they found only *Cyanidioschyzon* in aqueous environments.

Near-surface mineral deposits formed by the alteration of rocks by acidic waters include kaolinite, alunite, and quartz (Livo *et al.,* 2007), and hydrous ferric oxides, sulfur, goethite, and jarosite (Inskeep and McDermott, 2005; Jones and Renaut, 2007). These deposits are recognized as candidates for preserving biosignatures.

### 3.4. Bicarbonate springs

**FIG. 4.**
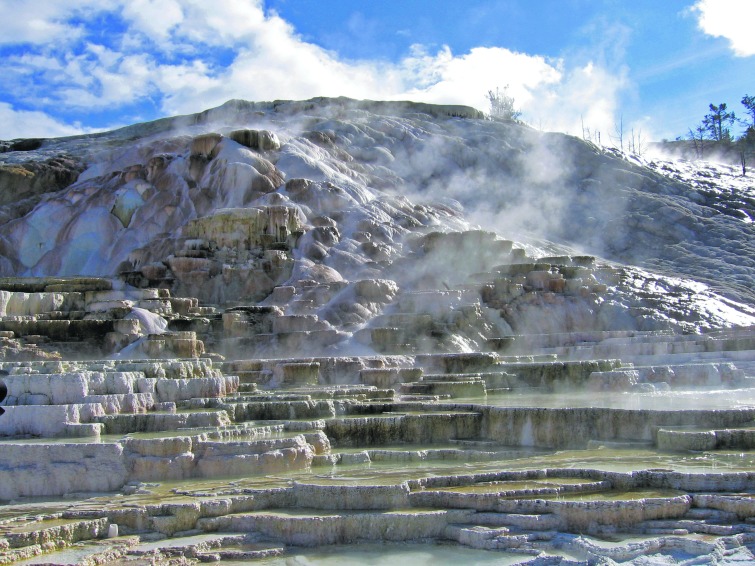
Palette Spring travertine deposits, Mammoth Hot Springs, Yellowstone National Park, USA.

On the northern margin of YNP at Mammoth there are large accumulations of travertine resulting from the emergence of deeply circulating spring waters that have been in contact with Paleozoic limestones and anhydrites (*e.g.,* Fig. 4). Fouke *et al.* (2000) described five depositional facies, as follows:

Spring waters are expelled in the vent facies at 71 to 73°C and precipitate mounded travertine composed of aragonite needle botryoids. The apron and channel facies (43–72°C) is floored by hollow tubes composed of aragonite needle botryoids that encrust sulfide-oxidizing *Aquificales* bacteria. The travertine of the pond facies (30–62°C) varies in composition from aragonite needle shrubs formed at higher temperatures to ridged networks of calcite and aragonite at lower temperatures. Calcite ‘ice sheets’, calcified bubbles, and aggregates of aragonite needles (‘fuzzy dumbbells’) precipitate at the air–water interface and settle to pond floors. The proximal-slope facies (28–54°C), which forms the margins of terracette pools, is composed of arcuate aragonite needle shrubs that create small microterracettes on the steep slope face. Finally, the distal-slope facies (28–30°C) is composed of calcite spherules and calcite ‘feather’ crystals. (Fouke *et al.,* 2000)

Fouke *et al.* (2000) conducted an extensive chemical and carbon and sulfur stable isotope study and concluded that, despite the presence of diverse microbial communities, abiotic factors such as degassing of CO_2_ are the predominant controls of the composition of the travertine. Evidence of microbial communities is preserved in the detailed fabric of the travertine, rather than in its chemistry.

Bacterial communities in spring outflow channels at Mammoth Hot Springs are distinctly partitioned between travertine depositional facies (Fouke *et al.,* 2003). A survey of 16S rRNA genes in these communities revealed that these sequences exhibited <12% similarity between each of the depositional facies. This partitioning of bacteria in these outflow channels apparently reflects systematic physical and chemical changes along the channels. Accordingly, the travertine facies might accurately indicate the composition of the bacterial communities as well and the morphology and chemical processes of travertine deposition (Fouke *et al.,* 2003).

### 3.5. Iron springs

**FIG. 5.**
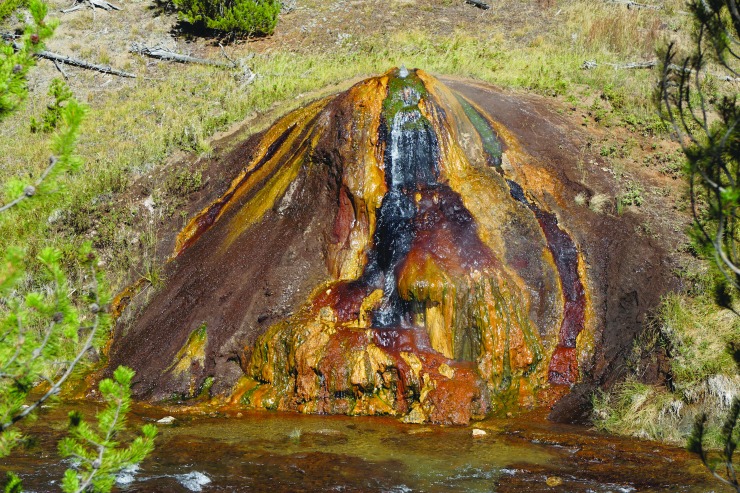
Chocolate Pots Spring, Yellowstone National Park, USA.

Chocolate Pots spring (Fig. 5) creates abundant flocculant iron oxide deposits that harbor microbial communities and could provide important insights about Precambrian iron-rich sedimentary deposits and ancestral phototrophic ecosystems (Walter, 1972; Parenteau and Cady, 2010). The slightly acidic (pH 5.7–5.9) anoxic waters have moderate to low chloride concentrations that are interpreted to derive from alkaline chloride waters that have mixed with acid-sulfate steam condensate waters that leached rhyolitic welded ash flow tuffs and flows (Parenteau and Cady, 2010), yielding abundant Fe^2+^ and Mn^2+^ solutes. Parenteau and Cady (2010) documented suites of biosignatures and associated features in higher-temperature *Synechococcus*-Chloroflexi mats and lower-temperature *Oscillatoria* mats. They observed ferrihydrite and traces of Ca and Mn oxide phases, and elevated organic carbon contents (0.8–2.9%). Mineral precipitates encrusted microbial rods, filaments, and condensed strands of extracellular polymeric substances. *Synechococcus* rods appeared to contribute to the formation of stromatolitic fabrics. Ferrihydrite-encrusted *Oscillatoria* filaments created characteristic dendritic structures. The encrustation by iron colloids of *Synechococcus,* Chloroflexi, and *Cyanothece minervae* cells favored their ultimate preservation as microfossils. Permineralization of *C. minervae* cells by ferrihydrite was observed deeper in the mats, indicating their ultimate potential preservation in goethite, hematite, or nontronite phases. Mineral deposits associated with iron-rich springs hold considerable potential as targets of exploration for their fossil records.

## 4. Taphonomic Processes

Taphonomy is the study of the sequence of processes that affect the remnants of organisms as they are transformed from the living state to the fossil record (see, *e.g.,* Allison and Briggs, 1991). These processes include scavenging, decomposition, physical fragmentation, transport, diagenesis (*i.e.,* postdepositional chemical degradation and physical compaction), and metamorphism (caused by elevated temperatures and pressures). Agents that attenuate these processes can thereby enhance the retention of biological information in fossils. For example, rapid sedimentation reduces the time available for scavenging, and diagenesis can limit the access of fluids that promote decomposition. Entombment by mineral precipitation can achieve this and also reduce fragmentation, transport, and physical compaction of biological remains.

The Rhynie Chert is a Devonian-age siliceous sinter deposit in Aberdeenshire, Scotland, that demonstrates in detail the effects of taphonomy on a freshwater community of flora and fauna (*e.g.,* Trewin, 1996). Degrees of preservation range from exquisitely retained three-dimensional internal anatomy of organisms that were completely silicified soon after death, to coalified laminae that were not entombed in silica until well after their decay and burial. The fidelity of preservation depended principally upon the extent of cellular decay prior to fossilization, and the timing and extent of silica permineralization and petrifaction (Trewin, 1996).

Whereas anatomical details can illustrate the fidelity of preservation of multicellular flora and fauna, the diversity of sinter microfacies can illustrate the preservation of hot spring microbial communities. Siliceous sinters in YNP, Wyoming, and Steamboat Springs, Nevada, USA, provide contrasting examples of the taphonomy of microbial communities. Hinman and Walter (2005) classified these sinters according to their synoptic relief and proposed biogenicity. At Artist Point, YNP, they identified the following microfacies: *low-relief structures (<2 mm relief)*—mound armor, stratiform geyserite, streamer fabric, and stratiform stromatolites; *moderate- to high-relief structures (2 mm to <10 cm)*—columnar geyserites and columnar stromatolites (“generic” columnar, columnar with ridges and spines, and columnar with domes); and *high-relief structures (>10 cm)*—spicules, which were relatively uncommon. In contrast, in the Plio-Pleistocene-age (pre-Lousetown) sinters at Steamboat Springs, Nevada, USA, Hinman and Walter (2005) identified only the stratiform and generic columnar stromatolite microfacies, and the streamer fabric microfacies. These sinters occur amid successions of andesitic basaltic lava flows, so they had experienced more elevated temperatures and different hydrological regimes than the deposits at YNP. Accordingly, most microfacies were obliterated. Hinman and Walter (2005) concluded as follows:

Differences in diagenetic histories related to depositional environment, water chemistry, and/or subsequent burial must account for the loss of textural evidence between the diagenetic stage represented by Artist Point sinters and that of Steamboat Springs sinters. Hence, early postdepositional history affects both the likelihood and quality of microfossil preservation.

Jones *et al.* (2001) examined silicified sinters around geysers and hot springs of North Island, New Zealand. Although in some cases microfacies fabrics could be interpreted as biogenic, the authors observed that organic matter and most cellular-scale features necessary to identify specific extant taxa were typically absent. For example, they proposed that the silicification process often obscures the presence of microbial sheaths and/or it significantly alters the apparent size of the microorganisms. They concluded as follows: “Direct comparisons [of modern versus] ancient silicified microbes should … be approached with caution until the taphonomic processes that control their early silicification, and the subsequent diagenesis of the opaline sinter to quartz (*cf.* Walter *et al.,* 1998; Campbell *et al.,* 2001), are understood fully.”

Orange *et al.* (2013) investigated these early silicification processes by simulating in the laboratory the splashing and evaporation of silica-rich waters in terrestrial hot springs. They examined silica precipitation as a result of cooling, evaporation, and pH change. They contrasted the results between experiments with and without the presence of the unicellular cyanobacterium *Synechococcus elongatus*. In the absence of the cyanobacterium, dense, finely laminated deposits formed, comparable to those formed in natural springs at temperatures above those that cyanobacteria can tolerate. In the presence of the cyanobacterium, nanometer-scale silica spheroids precipitated on the cell walls and in the enveloping mucous (EPS), thus preserving the cell morphology, as seen in natural sinter deposits. The results varied with the silica concentrations in the solutions—high concentrations resulted in silica precipitation directly in the supernatant and settling and entombment of the cells. Under all the experimental conditions, the cyanobacterial cells were “fossilized.” Westall *et al.* (1995) conducted similar experiments but without evaporation to elucidate the processes of fossilization in deep-sea environments; they demonstrated that some bacteria, and also fungi and diatoms, can be preserved.

Hydrothermal activity in cold surface conditions can enhance fossil preservation (Fox-Powell, 2018). “Cryogenic opal-A” (COA) precipitates within ice-bound brine channels in Icelandic hot springs. The surfaces of COA particles were populated with bacterial and archaeal rods and coccoids, and particle interiors retained *Chloroflexus* filaments. Fox-Powell *et al.* (2018) also detected biomolecules and inorganic microbial metabolic products in COA. The authors concluded that fossil preservation in COA is highly relevant to the exploration of extraterrestrial environments where hydrothermal activity has occurred under freezing conditions, for example on Mars and Enceladus.

## 5. Terrestrial Thermal Springs in the Geological Record

Thermal spring deposits have been documented in rock successions ranging in age from the Cenozoic (0–66 Ma) back to the oldest well-preserved sedimentary rocks on Earth (3.48 Ga) (Campbell *et al.,* 2015; Djokic *et al.,* 2017). Surprisingly, however, the pre-Cenozoic record seems very sparse. Why this should be so is not clear, but it may result from the deposits often being small and unfamiliar to most geologists. Similar terrestrial deposits such as fossil soils (palaeosols) are abundant (Retallack, 2001), suggesting that many spring deposits remain to be discovered. However, it is important to recognize that the paleontological record of life on Earth can be no better than the geological record, and that the further back in time we search the fewer the opportunities for preservation.

We focus here on the early (Mesozoic and older) record of life on Earth as a model in the search for life on Mars. To the best of our knowledge, the sedimentology and palaeontology of only three sets of thermal spring deposits of pre-Cenozoic Phanerozoic age have been studied in detail. They are the Jurassic examples in Patagonia (Argentina) and the Devonian examples in Queensland (Australia) and Scotland. These are all associated with “epithermal” gold and silver deposits, some of which are mined. Epithermal mineral deposits are relatively abundant in the rock record, again suggesting that there are many more spring deposits awaiting recognition and study. The springs of Patagonia and Queensland occur in what were extensional basins in back arc settings, while those of Scotland are in an extensional basin associated with strike-slip faults. All are associated with rhyolitic and andesitic volcanism (Cunneen and Sillitoe, 1989; White *et al.,* 1989; Rice *et al.* 2002; Guido and Campbell, 2009, 2011). A hot spring environment is inferred but not directly observed for a superbly preserved community of fossil ferns, fungi, and arthropods in the Jurassic of Sweden (McLoughlin and Bomfleur, 2016); preservation in this deposit apparently even extends to cell nuclei and chromosomes (Bomfleur *et al.,* 2014).

Remarkably, there seem to be no convincing records of terrestrial hot spring deposits of Proterozoic age. The 2.2 Ga Bartle Member of the Yerrida Basin in Western Australia is a strong candidate, but more work is required (Pirajno and Grey, 1997). For the Archean, the only convincing record is from a 3.48 Ga unit in the Pilbara Craton (Australia). Deposits from possible hot seeps occur in a 3.33 Ga succession in the Barberton Mountainland (South Africa). These two are described below. An additional possible example occurs in a very poorly outcropping succession at Kanowna in the Yilgarn Block in Western Australia, but at present the evidence is very limited (Grey, 1981).

### 5.1. Deseado Massif, Patagonia

In Patagonia, the Deseado Massif is a geological province characterized by extensive Jurassic volcanic and related rocks including rhyolites and minor andesites and dacites. During the Jurassic, extension, magmatism, and a high thermal gradient in a back-arc basin produced volcanics and related mineralization in the Massif, including economic gold- and silver-bearing epithermal deposits and at least 23 hot spring sites. The hot spring deposits comprise mostly travertines (carbonates), some siliceous sinters, and related cherts that are hosted in tuffs, breccias, and reworked volcaniclastic sediments within fluviolacustrine settings over a 230 × 230 km area. The distribution of the epithermal deposits is structurally controlled on a series of major faults (Guido and Campbell, 2011).

**FIG. 6.**
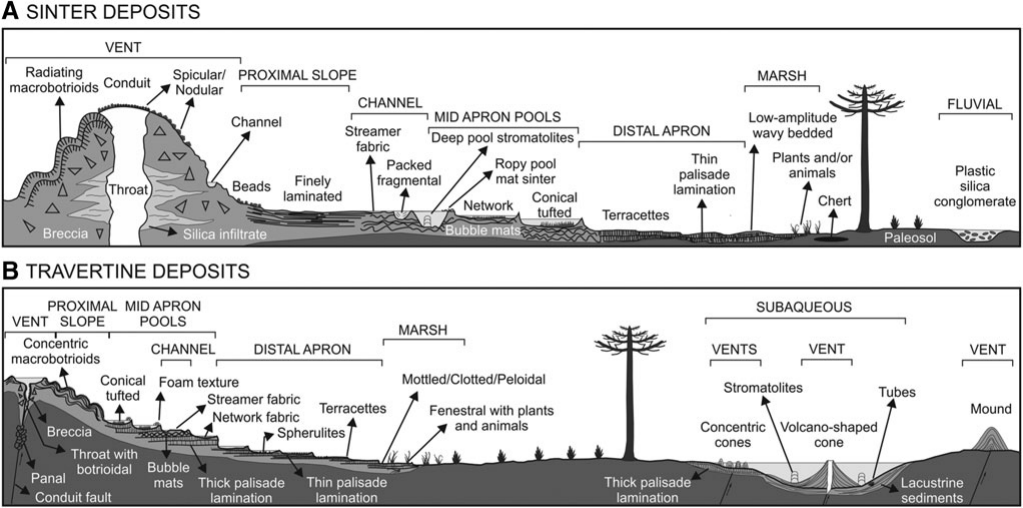
Diagrammatic representation of the Jurassic spring deposits of Patagonia. Reproduced with permission from Guido and Campbell (2011) and updated by those authors.

The five spring localities that Guido and Campbell (2011) studied in detail are closely associated with lacustrine and fluvial paleoenvironments that represent pauses in explosive volcanic activity. Spring activity lasted about 20 million years, and the individual deposits are several hundred meters to several kilometers in maximum lateral extent. The palaeoenvironments range from vent mounds to channels to marshes which by analogy with currently active springs in the Taupo Volcanic Zone of New Zealand (Jones *et al.* 1997a, 1997b; Jones and Renaut, 2012; Schinteie *et al.,* 2007) and YNP in the USA (Weed, 1889; Walter *et al.* 1972, 1976; Walter, 1976b; Braunstein and Lowe, 2001; Lowe *et al.,* 2001) would represent temperature gradients from the boiling point of water to ambient atmospheric temperatures. Depending on the pH and chemistry of the waters, the mineralogy of the spring deposits is either chert or travertine. Up to 20 sedimentary facies can be recognized in detail (Fig. 6). The deposits range in thickness from a few centimeters to a few meters.

Stromatolitic fabrics are abundant, especially in middle slope to distal apron settings. Such fabrics are commonly but not exclusively constructed by photosynthetic microorganisms (principally cyanobacteria) that develop a variety of growth morphologies (Handley and Campbell, 2011). Conical tufts and stratiform palisade fabrics are comparable with those constructed by filamentous cyanobacteria in YNP (Walter *et al.,* 1976). Microbial biofilms of Bacteria and Archaea also are present in modern hot springs, especially in high-temperature vent areas, but there is no cellular preservation once the enclosing silica recrystallizes to chert (*e.g.,* Cady and Farmer, 1996; Schinteie *et al.,* 2007). In cool sites distant from the vents, plant fossils as large as trees are common (Channing and Edwards, 2009, 2013).

### 5.2. Drummond Basin, Queensland

This back-arc basin is of Devonian to Carboniferous age. Seventeen epithermal mineral deposits with gold and silver have been recognized, and seven are known to be associated with hydrothermal sinters (Cunneen and Sillitoe, 1989; White *et al.,* 1989). All the sinters are siliceous. They are up to several hundred meters in lateral extent and up to two meters thick. They are interbedded with tuffs, conglomerates (lahars?), sandstones, and siltstones, in dominantly volcanic units of rhyolite, trachyte, and dacite. We have studied two of the sinters in detail (Walter *et al.,* 1996, 1998). The age is not well established but is most likely Late Devonian, based on characteristic plant fossils.

**FIG. 7.**
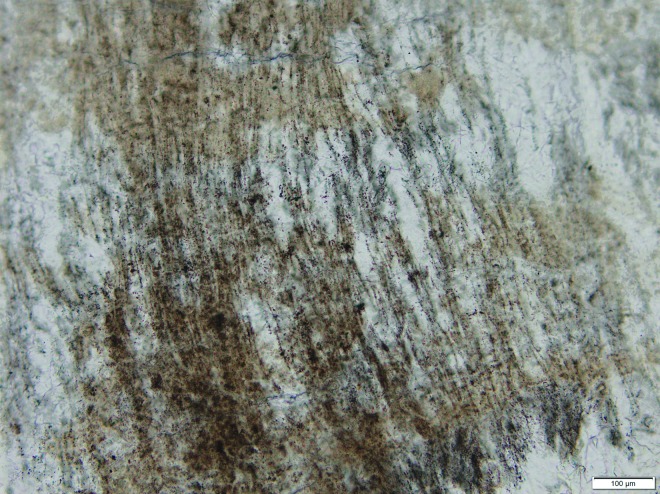
Preserved microbial filament molds from Devonian sinter in the Drummond Basin, Queensland, Australia (Walter *et al.,* 1996, 1998). Scale bar 100 μm. Photograph of “Wogegong North” sinter (Walter *et al.,* 1996) by Andrew Gangadine.

Thirteen different microfacies have been recognized, ranging from vent deposits through channels and interfluves to distal marshes. These are much the same as the Jurassic examples described above and in Figs. 1–6. As with modern analogs such as those in YNP and the Taupo Volcanic Zone, the sinters that can be inferred to have formed at less than 73°C are stromatolitic and frequently contain filamentous microfossils that are probably of cyanobacterial origin (Fig. 7). Abundant herbaceous lycopsid plant fossils are encrusted by microbial overgrowths and occur in the interfluve and marsh facies.

### 5.3. Rhynie Basin, Scotland

The Rhynie and Windyfield Cherts are famous for their superbly preserved fossils, first described by Kidston and Lang (1917). Neither unit outcrops, so information comes from float specimens, trenches, and more recently from shallow drill holes (Trewin, 1996; Rice *et al.,* 2002; Trewin *et al.,* 2003). These Early Devonian fossils represent the oldest known well-preserved terrestrial ecosystem. Included are early plants, algae, fungi, lichens, cyanobacteria, and arthropods. The plant fossils in the Rhynie Chert are so well preserved that they set a benchmark in the history of the metaphytes. Preservation extends to the cellular level as permineralized organic matter. While morphological preservation is superb, thermal alteration has proven too intense for molecular biomarkers to be preserved; nonetheless, the presence of aliphatic and aromatic hydrocarbons has been determined (Abbott *et al.,* 2017). A range of microfacies comparable to those described above for younger deposits is observed, in isolated specimens and cores. The very extensive literature on this deposit is summarized by Edwards *et al.* (2017).

### 5.4. Barberton greenstone belt, South Africa

The 3.33 Ga Josefsdal Chert in the Barberton Mountainland of South Africa was deposited in an intermittently exposed marine coastal volcanic setting. Volcanic sediments were suffused by silica-rich hydrothermal fluids that preserved a record of what are interpreted as phototrophic, chemolithotrophic, and chemo-organotrophic microbial communities (Westall *et al.,* 2015, 2018). The chert is up to 30 m thick and contains some units that may have formed in surface hydrothermal seeps. No geyserite or structured stromatolites are recorded, although there are probable fossil biofilms.

### 5.5. Pilbara Craton, Western Australia

The 3.48 Ga Dresser Formation was deposited within a volcanic caldera affected by voluminous hydrothermal fluid circulation. There are two horizons of silicified sedimentary rocks alternating with pillowed to massive metabasalts. The formation is exceptionally well preserved for its age, exhibiting low-strain and low-grade metamorphism, specifically prehnite–pumpellyite to lower greenschist facies. The lowest sedimentary horizon of the Dresser Formation is a fossiliferous unit exposed for 14 km along the eastern flank of the North Pole Dome. It consists of gray and white layered chert with subordinate volcaniclastic sandstone, jasplitic chert, bedded carbonate, and stromatolites. Underlying hydrothermally altered basalts are transected by a dense network of silica (microquartz) ± barite ± pyrite ± organic matter–bearing hydrothermal veins. The veins formed contemporaneously with sediment accumulation, as they disperse into but do not pass through the sedimentary units. The sedimentary unit contains finely laminated chert that is interpreted as geyserite and a suite of biosignatures that include stromatolites, putative microfossils, and carbon and sulfur isotopic patterns consistent with biological activity (Djokic *et al.,* 2017).

## 6. Ancient Springs on Mars

The extent and significance of hydrothermal activity on Mars have been made increasingly apparent by a progression of key discoveries since 1971. The 1971 Mariner 9 orbiter revealed that extensive lava plains and enormous volcanoes cover vast expanses of the surface (*e.g.,* Carr, 1973). Mariner 9 also discovered prominent river channels in several regions, indicating that large volumes of water carved deep valleys that, in some cases, extended thousands of kilometers. The 1976 Mars Viking missions corroborated and extended these early discoveries and also identified linkages between volcanism and aqueous activity (Carr, 1996, 2006). For example, Dao Vallis is a large springlike feature located on the southern slope of the volcano Hadriacus Mons and extending to the southwest into Hellas Planitia. The large volume of water that carved this feature very likely arose when hot magma melted frozen ground ice (Farmer, 1996; Carr, 2006). By the late 1970s, Mars missions had confirmed the widespread presence of volcanism and abundant crustal water, components that are essential for sustaining hydrothermal activity (*e.g.,* Gulick, 1998). Subsequent observations increased the number and broadened the distribution of documented features that morphologically resemble thermal spring deposits. For example, in Arabia Terra, light-toned structures resembling spring mounds are aligned along an extensive fracture system that conceivably facilitated the ascent of warm fluids (Allen and Oehler, 2008). Layered to massive light-toned-deposits are widely distributed in Valles Marineris, several large craters, and chaotic terrains (Rossi *et al.,* 2008). Rossi *et al.* argue the following: “Stable volcano-tectonic settings, such as those typical on Mars, are compatible with a large-scale, long-term, multistage formation of spring deposits.”

Orbital spectroscopic observations have strengthened substantially the evidence for hydrothermal activity. The CRISM VIS-NIR instrument on the Mars Reconnaissance Orbiter (MRO) detected hydrated silica deposits near the summit and on the flanks of volcanic cone in the Syrtis Major caldera complex (Skok *et al.,* 2010). Michalski *et al.* (2017) documented ancient (*ca.* 3.8 Ga) massive deposits in the Eridiania region that contained “…saponite, talc-saponite, Fe-rich mica (for example, glauconite-nontronite), Fe- and Mg-serpentine, Mg-Fe-Ca-carbonate and probable Fe-sulfide that likely formed in a deep water (500–1,500 m) hydrothermal setting.” In the Late Hesperian Noctis Labyrinthus complex, several 100 m of stratified deposits were altered to form Fe-smectite and Fe-sulfates by solutions interpreted to have derived from groundwater and magmatic sulfur (Thollot *et al.,* 2012). Minerals and mineral assemblages indicating hydrothermal aqueous alteration or low-grade metamorphism tend to be heterogeneously distributed in cratered terrains (Ehlmann *et al.,* 2009). In addition to volcanism, large impacts also can provide energy to drive hydrothermal activity (Osinski *et al.,* 2013).

Discoveries by the Mars Exploration Rover Spirit in an ancient volcanic hydrothermal setting in Gusev Crater illustrate the significance that thermal springs hold for astrobiology. Opaline silica deposits arose from a combination of fumarolic alteration of basaltic bedrock and the deposition of sinter from near-neutral pH alkali chloride waters (Squyres *et al.,* 2008). Some deposits feature knobby digitate structures interpreted as potential biosignatures (Ruff *et al.,* 2011). Morphologically similar structures in hot spring siliceous sinter at El Tatio, Chile, are microstromatolites that have entombed microbial filaments (Ruff and Farmer, 2016).

## 7. Summary

Terrestrial thermal springs are abundant in currently active volcanic regions on Earth and are known throughout the geological record back to the oldest well-preserved rocks at 3.48 Ga in the Pilbara region of Western Australia. It is likely that many more ancient deposits exist than are currently recognized. The diverse chemistries of modern thermal springs are determined by the lithologies, temperatures, and pressures that the heated waters encounter at depth and during their ascent to the surface. Water temperatures range up to boiling, and their pH values range from highly acidic to alkaline. Solutes and chemical redox gradients in the emerging spring waters provide nutrients and sources of redox energy for microorganisms. The springs can precipitate siliceous sinter (opal-A: SiO_2_·nH_2_O), travertine (CaCO_3_), iron oxyhydroxides, and other phases. Surface and near-surface waters harbor diverse microbial communities. At temperatures below 73°C in neutral to alkaline waters, cyanobacteria are abundant and form distinctive stromatolites that can be rapidly mineralized. Bacteria and archaea are also abundant at higher temperatures and in more acidic waters and seem to contribute to the morphogenesis of the mineral deposits proximal to spring vents. Thus diverse microbial communities are morphologically preserved into the rock record. Such springs and their deposits can be expected on other terrestrial planets, and several candidates have been detected on Mars. Martian sinters may well contain a record of microbial life on that planet, if such life ever existed there. These sinters are obvious targets for future exploration. The lesson from Earth is that spring deposits can contain a rich record of life.

## Abbreviations Used

COA: cryogenic opal-A
YNP: Yellowstone National Park
